# Development of GO/Co/Chitosan-Based Nano-Biosensor for Real-Time Detection of D-Glucose

**DOI:** 10.3390/bios12070464

**Published:** 2022-06-27

**Authors:** Dong Sup Kim, Xiaoguang Yang, Ja Hyun Lee, Hah Young Yoo, Chulhwan Park, Seung Wook Kim, Jinyoung Lee

**Affiliations:** 1 Department of Green Chemical Engineering, Sangmyung University, 31 Sangmyungdae-Gil, Dongnam-Gu, Cheonan 31066, Korea; wavekds@korea.ac.kr; 2 Department of Chemical and Biological Engineering, Korea University, 145, Anam-Ro, Seongbuk-Gu, Seoul 02841, Korea; yangxg83@korea.ac.kr; 3 E & S Healthcare Ltd., Suite N313, 11-3, Techno 1-ro, Yuseong-gu, Daejeon 34015, Korea; 4 Department of Convergence Bio-Chemical Engineering, Soonchunhyang University, 22, Soonchunhyang-ro, Asan-si 31538, Korea; jhlee84@sch.ac.kr; 5 Department of Biotechnology, Sangmyung University, 20, Gongjimun, 2-Gil, Jongno-Gum, Seoul 03016, Korea; y2h2000@smu.ac.kr; 6 Department of Chemical Engineering, Kwangwoon University, 20 Kwangwoon-Ro, Nowon-Gu, Seoul 01890, Korea; chpark@kw.ac.kr

**Keywords:** GO/Co/chitosan, nano-biosensor, real-time detection, D-glucose, glucose oxidase

## Abstract

Electrochemical nano-biosensor systems are popular in the industrial field, along with evaluations of medical, agricultural, environmental and sports analysis, because they can simultaneously perform qualitative and quantitative analyses with high sensitivity. However, real-time detection using an electrochemical nano-biosensor is greatly affected by the surrounding environment with the performance of the electron transport materials. Therefore, many researchers are trying to find good factors for real-time detection. In this work, it was found that a composite composed of graphite oxide/cobalt/chitosan had strong stability and electron transfer capability and was applied to a bioelectrochemical nano-biosensor with high sensitivity and stability. As a mediator-modified electrode, the GO/Co/chitosan composite was electrically deposited onto an Au film electrode by covalent boding, while glucose oxidase as a receptor was immobilized on the end of the GO/Co/chitosan composite. It was confirmed that the electron transfer ability of the GO/Co/chitosan composite was excellent, as shown with power density analysis. In addition, the real-time detection of D-glucose could be successfully performed by the developed nano-biosensor with a high range of detected concentrations from 1.0 to 15.0 mM. Furthermore, the slope value composed of the current, per the concentration of D-glucose as a detection response, was significantly maintained even after 14 days.

## 1. Introduction

Nano-biosensors are analytical devices composed of biological materials with physicochemical detectors. Due to the capacity to sense many biological reactions, a nano-biosensor can be applied in various areas, such as medical care, environmental protection, food safety, and even industrial bioprocessing monitoring [1,2,3]. As a major type of nano-biosensor, the amperometry biosensor has been designed based on bio-electrocatalysis, which converts biochemical reactions to electrical signals [2,3,4]. The specificity of bio-catalysis could be utilized for an amperometry nano-biosensor for a specific target detection. In various sensing applications, the glucose nano-biosensor has attracted the attention of researchers because it is closely associated with our daily lives. Due to its cost effectiveness and rapid analysis, nano-biosensors have been shown to have higher sensitivity and stability than traditional detection methods [5].

Many applications of a nano-biosensor have been reported in the medical and food areas [2], while the complex environment of a sensing target requires a nano-biosensor to have specificity. The specificity of a nano-biosensor could be achieved by enzymatic reaction using glucose oxidase (Gox) for the real-time detection of glucose. The electrical signal from this enzymatic redox reaction could detect glucose contained in the solution samples [3,4]. Glucose can be effectively coupled to Gox on the electrode surface with Gox immobilization. The principle of a bioeletrochemical nano-biosensor is similar to the concept of biological fuel cells using a biomaterial as fuel. Redox reactions by Gox on metal electrodes can convert glucose to gluconic acid with electron power generation [1]. In this process, the electrode as a key factor could directly influence the performance of a nano-biosensor, while the shape modification of electrodes is required to overcome the limitation of current nano-biosensors. A film-type electrode has several advantages for nano-biosensor applications compared to a traditional electrode in stick form [5,6].

A proper electron transfer mediator immobilized on an electrode significantly affects the bioeletrochemical detection of nano-biosensors. Generally, layer- or matrix-structured materials such as graphite-based nanocomposite films are employed for electrode modification as mediators to improve electron transfer and to finally enhance electrochemical sensing [7,8,9]. In our previous study, a mediator consisting of graphite oxide, cobalt, and chitosan was developed [8]. It could significantly improve the electro-catalytic activity on an Au electrode [8,10]. In addition, chitosan is a natural biopolymer that aids in enzyme immobilization on electrodes, which is a crucial process in the establishment of an electron transfer facility. The electron transfer, mechanical, and immobilization properties of chitosan are influenced by its solubility, while chitosan can even exhibit hydrogel formation and viscoelastic behavior. The slightly acidic pH of chitosan solutions positively influences its rheological and morphological properties, as well as its electrochemical performance [11]. Therefore, the use of chitosan as a mediator could be another effective pathway to improve the sensing performance of a bioeletrochemical nano-biosensor. Furthermore, Cobalt with chitosan is one of the heavy-metal materials that has good performance regarding electron transfer because it contains ferromagnetism and pseudocapacitance. The reaction of a complex salt using cobalt chloride (CoCl_2_) forms cobalt hydroxide (Co(OH)_2_). Cobalt hydroxide has well-defined electrochemical redox activity [12]. Cobalt has been utilized in alkaline batteries, fuel cells and capacitors and is a promising material for electrodes, where the GO surface is modified by cobalt [13]. The introduction of a GO/Co composite contributed to an enhancement of power density, thereby increasing the affinity between enzymes and electrodes.

In this study, the electron transfer mediator immobilized on a film-type Au electrode was modified by GO/Co/chitosan deposition in the process of manufacturing a bioelectrochemical nano-biosensor for the detection of D-glucose. The redox behavior and electrode transfer properties of the established electrode were determined by cyclic voltammetry (CV) and electrical impedance spectroscopy (EIS). Electrochemical measurements were carried out with a broad range of glucose concentrations to investigate the sensing mechanism [11,14]. The sensing performance of the developed GO/Co/chitosan-based nano-biosensor was confirmed for sensitivity and stability regarding the detection of D-glucose in various concentrations.

## 2. Materials and Methods

### 2.1. Chemicals

Graphite powder (synthetic < 20 µm), KMnO_4_, H_2_SO_4_, Cobalt (II) chloride hexahydrate (CoCl_2_·6H_2_O), chitosan with medium molecular weight, glucose oxidase (Gox; EC1.1.3.4, derived from Aspergillus niger), covalent bonding agents as N-(3-dimethylaminopropyl)-N’-ethylcarbodiimide hydrochloride (EDC), and N-hydroxysuccinimide (NHS) were purchased from Sigma-Aldrich reagent Co. (Saint Louis, MO, USA). Ammonium hydroxide (NH_4_·OH), anhydrous dextrose (98%, *w*/*v*), and potassium permanganate were purchased from Samchun Pure Chemical Company (Pyeongtaek-si, Korea). Deionized (DI) water with a resistivity of 18.1 MΩ·CM was produced using an ultra-pure water system (Pure UP90, Namyangju, Korea) purifier system.

### 2.2. Preparation of Electron Transfer Mediator and Immobilization Process of Glucose Oxidase on an Au Film Electrode

Gold (Au) film was approximately cut to have an area of 10 × 10 mm. It was then cleaned with alcohol for preparing an Au film electrode. The mediator consisting of GO/Co/chitosan was prepared as a mixed solution, which was then electro-deposited onto the surface of the Au film electrode according to a previous method [15,16]. After electro-deposition of GO/Co/chitosan, a layer of amino groups was prepared on the surface of the Au film electrode for further enzyme immobilization steps. Power Supply EV215 was used for deposition of the mediator at the film electrode with a voltage range of 10–50 V [16]. Gox was employed to establish a glucose biosensor. To evaluate the performance, the electrochemical detections of the biosensor using the developed film electrode were carried out with various conditions. These detections were analyzed with a VersaSTAT 3 electrochemical analyzer with three electrodes: working electrode, counter electrode and commercial Ag/AgCl electrode as a reference electrode.

**Scheme 1:**
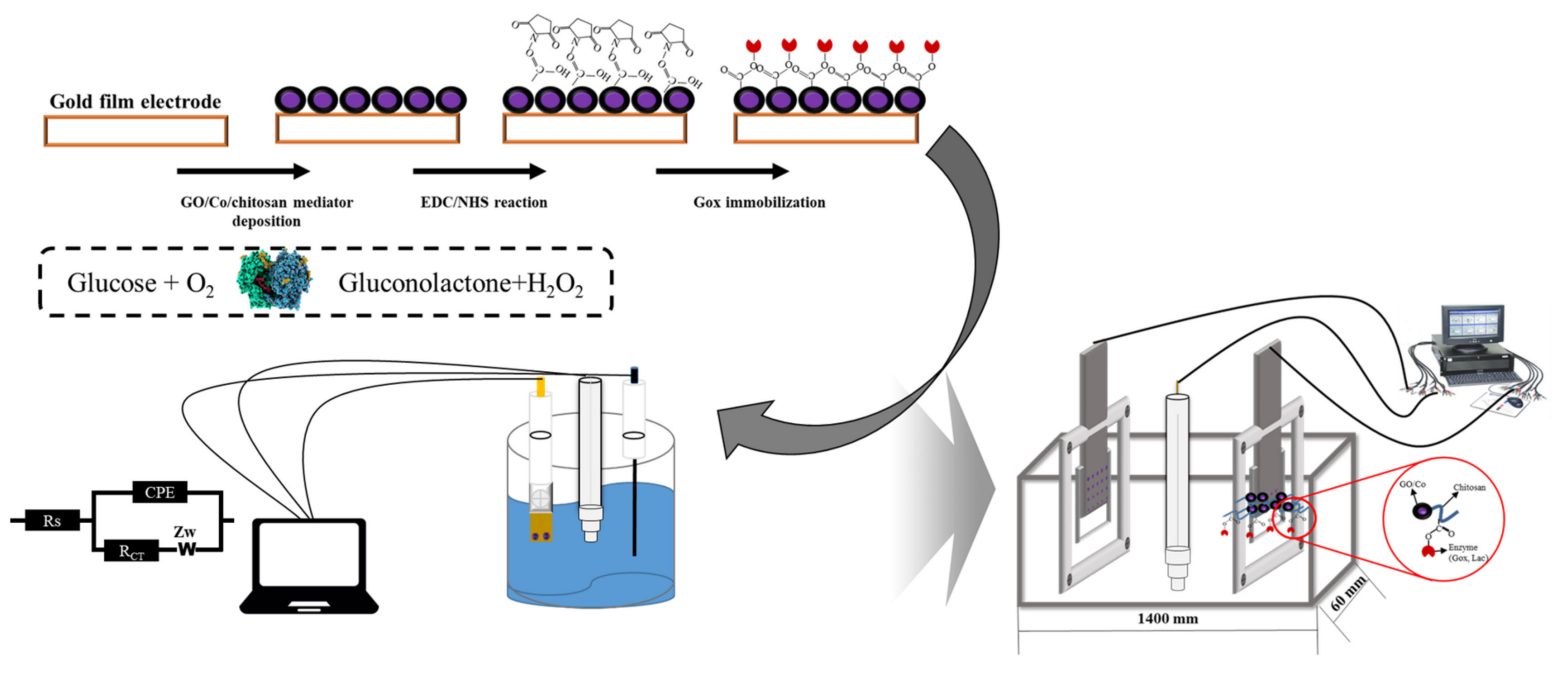
Immobilization process of GO/Co/chitosan-based nano-biosensor and bioelectrochemical system for sensor analysis.

Gox was immobilized on the mediator-modified electrode. Enzyme immobilization was carried out with 1 g/L of Gox in phosphate buffer (0.1 M, pH 7.0) containing 20 mM of EDC and 5 mM of NHS as covalent-bonding reagents. After 4 h of immobilization re-action at 4 °C, the modified electrode was stored at 4 °C for 4 h before utilization [14]. Both sides of the film electrode were treated with the same modification method mentioned above (Scheme 1). Both sides of the film electrode were treated with the same modification method. Scheme 1 shows the steps and chemical changes of the surface of the electrode modification, as shown above and to the left. The bottom left of Scheme 1 shows the ideal electrochemical reaction of the sensor system. The right side of Scheme 1 shows the sensor system established in this study.

### 2.3. Surface Analysis of Modified Electrode

The surface of the GO/Co/chitosan-modified Au film electrode was observed with a field emission–scanning electron microscope (FE–SEM). Energy dispersive X-ray spectrum (EDS) and elemental mapping images of the synthesized composite nanosheets of the film electrode surface were obtained at a high-vacuum mode using a FEI Quanta FEG 250 field FE-SEM with ThermoFisher Quantax EDS attachment. EDS was recorded with a FEI Quanta FEG 250. The mediator-modified Au film was observed by Fourier transform infrared spectroscopy (FT-IR, PerkinElmer, Inc., Waltham, MA, USA) to confirm functional groups on the electrode surface after modification.

### 2.4. Electrochemical Analysis of Developed Biosensor

The Gox/GO/Co/chitosan/Au film electrode developed in this study served as the working electrode of a three-electrode system, in which a bronze-based gold electrode and commercial platinum electrode were used as the working electrode and counter electrode, respectively. With various glucose concentrations (from 1 to 15 mM), cyclic voltammetry (CV) measurements were carried out in 0.1 M phosphate buffer from −0.8 to 0.2 V with a scan rate of 0.1 V/s. Impedance spectroscopy (EIS) was measured at a frequency from 0.01 Hz to 10 kHz.

To further study the performance of a developed film-type biosensor, glucose sensing of a commercial beverage was demonstrated by CV measurement. The sample of the commercial beverage was diluted 10 times with DI water. This real case CV was detected with the VersaSTAT 3 electrochemical analyzer as described previously [12]. Scheme 1 shows the immobilization process of a Gox/GO/Co/chitosan-based nano-biosensor and a bioelectrochemical system for sensor analysis.

## 3. Results and Discussion

### 3.1. Surface Analysis of GO/Co/Chitosan Modified Film-Typed Electrode

**Figure 1:**
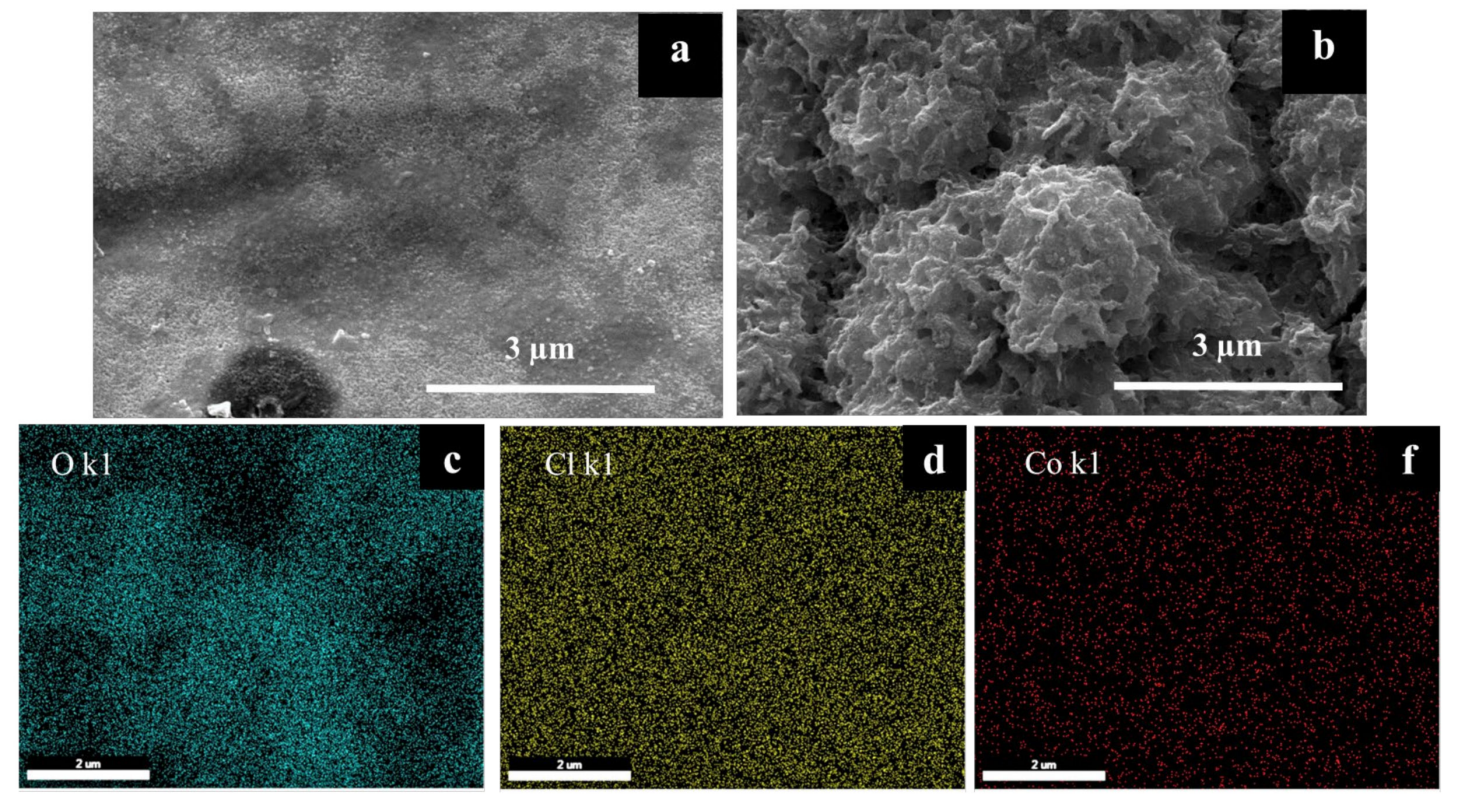
Surface analysis using FE-SEM and elemental mapping images of the electrode modified with GO/Co/chitosan in the developed biosensor: (**a**) bare electrode; (**b**) modified electrode with GO/Co/chitosan. Elemental mapping images of (**c**) O; (**d**) Cl; (**f**) Co; EDS analysis.

The surface of the Au film electrode modified with a GO/Co/chitosan composite with element distribution was analyzed by FE-SEM (Figure 1). According to the SEM images shown in Figure 1a, the GO/Co/chitosan composite was successfully distributed onto the Au film of the electrode surface with varying shapes of formation compared with those of the bare electrode (Figure 1b). Element compositions of this mediator are shown in Figure 1c,d,f confirming that the GO/Co/chitosan complex was formatted with effective compositions based on our previous study of this mediator structure [14]. The distribution of O in cyan color revealed elements O, Co, and Cl shown in sky blue, pink, and white, respectively. These SEM results indicated that the major elements of GO/Co/chitosan were uniformly distributed onto the Au film. The results also confirmed a uniform distribution of the mediator deposited onto the electrode surface. This uniform deposition is understood to be a complex layer covered on the surface to enhance electron transfer with on the entire area of the Au film, of which the effect modification was maximally developed.

**Figure 2:**
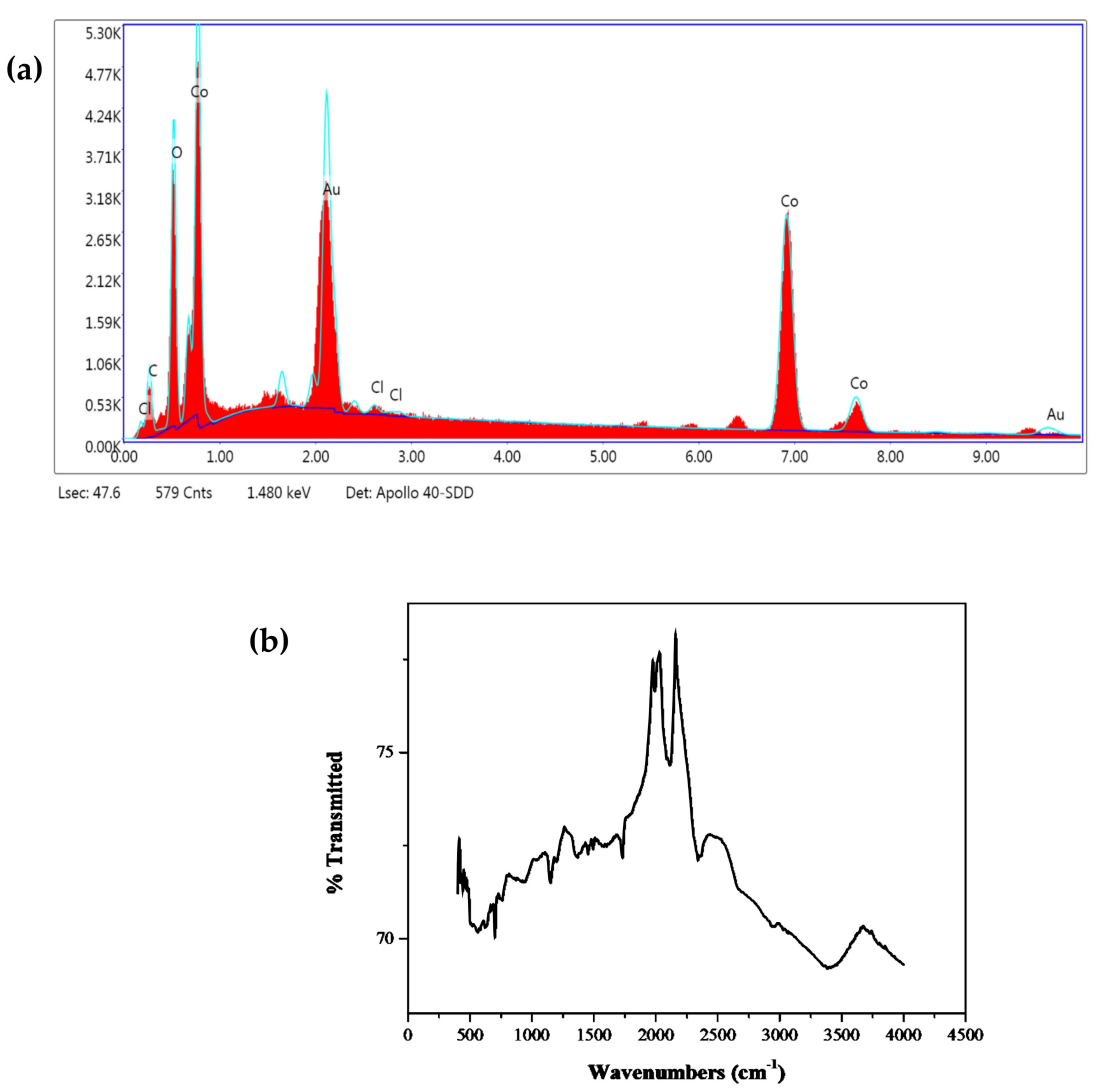
Analysis of the electrode modified with GO/Co/chitosan in the developed biosensor: (**a**) EDS of GO/Co/chitosan composite; (**b**) FT-IR spectra of GO/Co/chitosan composite. Detection condition is HV 10.00 KV; magnification at 1000×.

There are several picks of atom components at the surface of the modified electrode in GO/Co/chitosan, as shown in the EDS graphs in Figure 2a. After confirming the formation of the mediator deposition, the functional groups of GO/Co/chitosan were determined by FT-IR spectra to ensure the electrochemical properties of this complex modification (Figure 2b). According to previous reports on characteristic absorptions of chitosan, hydroxyl groups are usually shown in the range of 2800 to 2950 cm^−1^ with stretching vibration of around 3695 and 3073 cm^−1^ [17]. The FT-IR spectra of GO/Co/chitosan shown in Figure 2 revealed an absorption band of -OH group at 2876 cm^−1^ with stretching vibration from 3666 to 3023 cm^−1^, respectively. Meanwhile, the absorption peak of C-O-H stretching presented at 1201 cm^−1^, as shown in Figure 2, was associated with bridge-O-stretching reported at 1126 cm^−1^ [18]. The broad peak at 1149 cm^−1^ was ascribed to C-O stretching vibration of the C-O-H, CO-C, and CH_2_OH rings [19,20]. In addition, the bending vibration of the methyl group of chitosan was observed at about 1368 and 1482 cm^−1^, the spectrum of the chitosan complex was observed at 1595 cm^−1^, and the carbonyl C=O was reported to have been stretched at 1667 or 1746 cm^−1^ [21]. Similar results were found for the absorption band of C-H stretch, which remarkably shifted from 1383 to 1360 cm^−1^ after deposition of the chitosan complex. These changes of FT-IR results have been reported for a hybrid complex with two or more polymer mixtures, for which characteristic spectra peaks are influenced by the reflection of physical blends and chemical interactions [22]. The FT-IR spectra of the GO/Co/chitosan indicated a good miscibility of this mediator between chitosan and Co graphite. All active chemical properties remained after composite formation. In addition, functional groups of GO/Co/chitosan were confirmed after the complex was deposited onto the Au film as shown in Figure 2, thus ensuring modification effects on the electrode surface. The deposition process in this study could also be considered as a physical and chemical reaction. The confirmation of the functional group of complexes after deposition was necessary before using, which could be considered as confirmation of modified functions on the surface of the electron. This function confirmation indicated the target deposition. After confirming the composition, deposition, and functions of the complex, the modification process was completed.

### 3.2. Electrochemical Property of the Developed GO/Co/Chitosan-Based Nano-Biosensor

**Figure 3:**
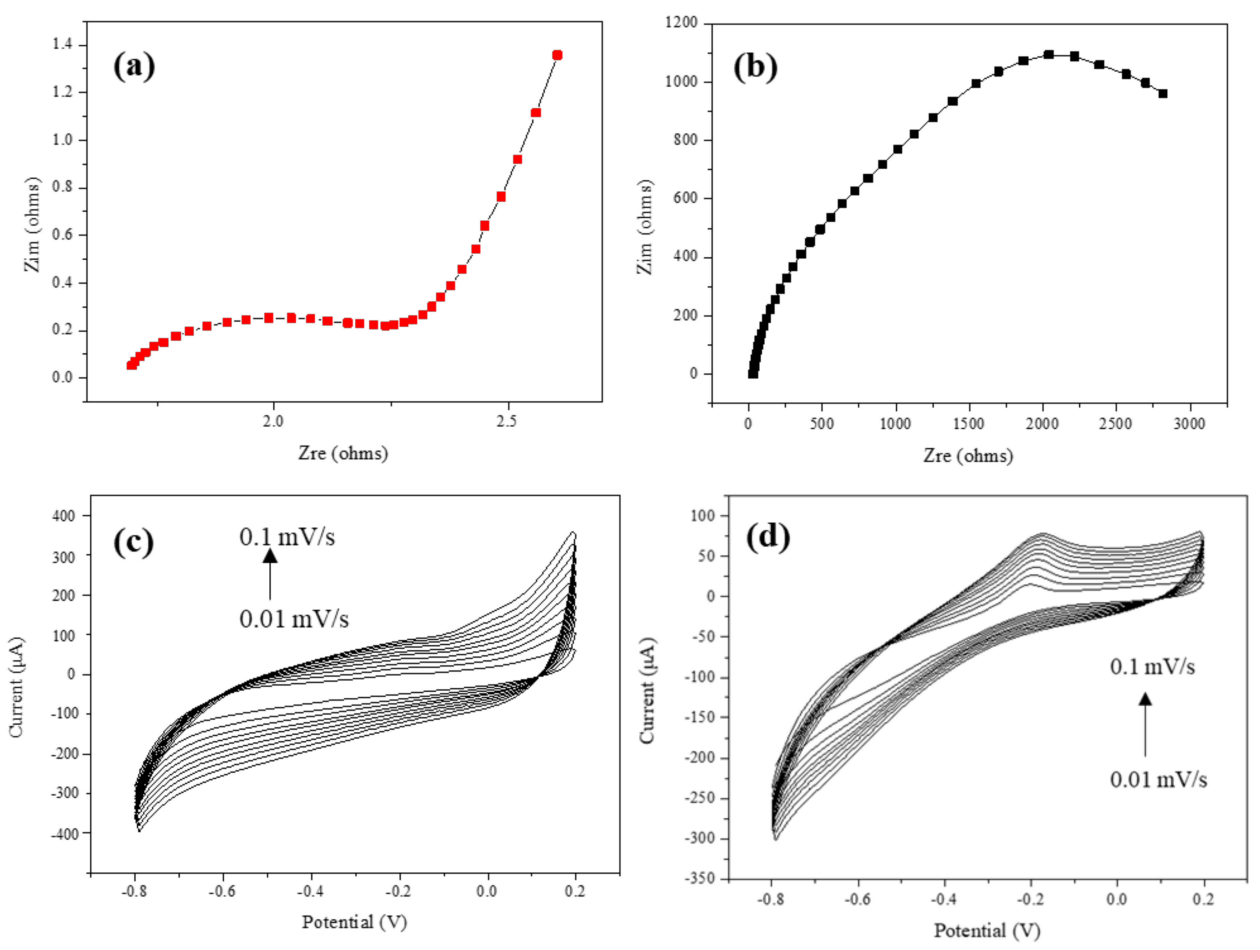
Electrochemical properties of the developed GO/Co/chitosan-based nano-biosensor. EIS measurement of the (**a**) GO/Co/chitosan electrode; (**b**) bare electrode. Cyclic voltammetry measurement following the use of D-glucose for glucose oxidase immobilization onto the modified Au electrode: (**c**) 1st day; (**d**) 14th day.

To establish a biosensor for glucose detection, Gox was immobilized on the mediator-modified Au film electrode. Nyquist plots of the Au bare and modified electrodes after enzyme immobilization were prepared to investigate the electrical properties of the prepared electrodes. EIS results of the modified electrode with immobilized Gox are magnified as a red curve compared with those of the bare electrode (Figure 3a,b). The semi-circle portion corresponds to a high-frequency region of electron transfer resistance (Rct), which could be an important parameter to evaluate the conductivity of an electrical material. The Rct value could be quantified based on the diameter of the semi-circle portion. It was calculated to be 2.3 Ω for the modified electrode with Gox immobilization and 2.8 kΩ for the bare Au electrode. The electron transfer resistance of the electrode was dramatically decreased after mediator modification and enzyme immobilization. The chitosan and polypeptide backbone of Gox showed some insulating properties. However, the GO/Co/chitosan was found to be a conductive poly-network coating on the Au film surface with excellent conductive properties of GO and cobalt chloride hexahydrate. The conductivity of the electrode after Gox was immobilized in the mediator matrix was significantly improved for an efficient electron transfer. With the confirmed results of Rct in EIS, electrode modification using GO/Co/chitosan composites and Gox immobilization was demonstrated to be suitable for a biosensor.

As a key factor of the biosensor, redox reaction with immobilized Gox on the electrode directly influenced electron productivity. Thus, it was necessary to determine the mechanism of Gox catalysis in this electrochemical application. Cyclic voltammograms (CVs) of the developed biosensor were obtained with designated potentials to investigate reversible and irreversible oxidation on the electrode. The CV results showed oxidation peaks from −0.2 to 0.2 V and reduction peaks from −0.8 to −0.6 V. The oxidase peaks of CV results for different D-glucose concentrations could be obviously distinguished, confirming that efficient redox reactions occurred on the Gox-immobilized electrode. D-glucose is oxidated to be gluconolactone, while flavin adenine dinucleotide (FAD) as a cofactor of Gox is reduced to be FADH_2_. The reduced FADH_2_ then releases two hydrogens, while hydrogen peroxide (H_2_O_2_) is produced. Finally, the H_2_O_2_ donates two protons and two electrons, as shown below:

Gox (FAD) + D-Glucose→Gox(FADH_2_) + Gluconolactone

Gox (FADH_2_)+O_2_→Gox (FAD)+H_2_O_2_

H_2_O_2_→O_2_ + 2H^+^ + 2e^−^

It was confirmed by CV cycle with a hydrogen peroxide pathway that the developed GO/Co/chitosan-based nano-biosensor enables the transfer of two elections using D-glucose. The formation of Co nanorods in the GO/Co/chitosan composite has been reported with significant promotion for electron transfer at the active site of Gox [9,13,22,23]. The electro-chemical performance of the GO/Co/chitosan-modified Au film electrode was evaluated by cyclic voltammogram (CV) with a scan rate from 0.01 to 0.1 mV/s with a 0.01 interval on the first day, as shown in Figure 3c. The results indicated that the redox reactions were completed while the peak point of this redox reaction was increased with an increasing scan rate. To further investigate the sensing ability of the modified film electrode, the amperometry response of the biosensor was detected with various glucose concentrations. With an increase in glucose concentration, the magnitude of the current signal was monotonically increased. This proportioned monotonically increasing relation of current and substrate containing was found until the glucose concentration was 15 mM. The current value could be clearly distinguished, demonstrating the feasibility of using an anodic peak current as a sensing parameter for this film-type biosensor. Moreover, the CV performance of this biosensor was carried out for 14 days after fabricating the film electrode, as shown in Figure 3d. Similar to the results on the first day, as shown in Figure 3c, redox reactions of the electrode surface were confirmed with an increasing scan rate for detection. Anodic peaks on the 14th day were lower than those of the first day. Such difference could be due to an increasing amount of reduced Gox after 14 days of fabrication. It has been reported that the active site of Gox (FADH_2_) could be widened during its reaction with glucose, in which hydrogen peroxide was selected as a substrate to combine with a reductive enzyme rather than to oxygen [24,25,26]. A decrease in H_2_O_2_ in the redox reaction system directly resulted in decreased reaction of its oxidation to O_2_, which significantly reduced electron generation from Gox catalysis. Therefore, with an established time, the current value of the oxidation peak might be decreased due to more Gox (FADH_2_) moving toward an inactive state which could not bind to glucose. To further investigate the sensing behavior of this biosensor, the CV results of the current peak values with various glucose concentrations were obtained. After 14 days of fabrication, a linear function of current peak was obtained: |Ic|P = 3.17 (μA/mM) × C + 0.044 μA (correlation coefficient: 0.9999). This high coefficient indicated a high accuracy of the biosensor for glucose detection. In addition, the sensitivity of this Au film electrode-based biosensor was determined to be 0.14 μA/mM‧cm^2^ with a detection limit of 2.7 mM. Both sensitivity and glucose-sensing limitation of this biosensor were significantly improved compared to the reported biosensors using immobilized Gox on the Au electrode [22,23,24]. This facilitation of sensing performance could be attributed to the mediator GO/Co/chitosan, which could improve both electron transfer of the electrode and enzyme immobilization stability.

**Figure 4:**
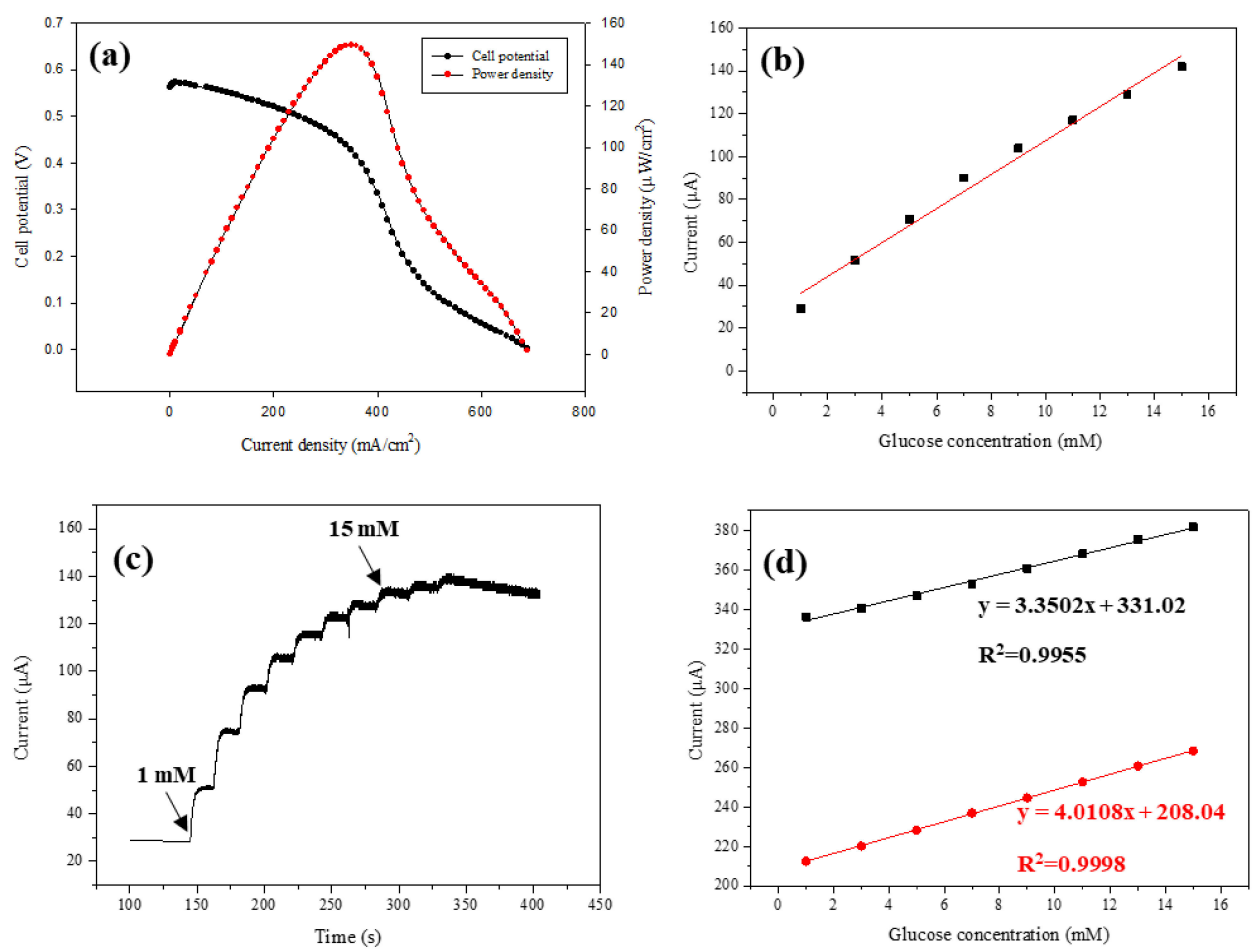
Biosensor capacity of the developed GO/Co/chitosan-based nano-biosensor. (**a**) Power output and cell potential versus current curves of the glucose oxidase modified electrode with GO/Co/chitosan at different external resistances compared with those of the bare electrode. (**a**) GO/Co/chitosan immobilized electrode. (**b**) Bare electrode; sensor capacity of the developed nano-biosensor. (**c**) Continuous real-time detection test. Inset of D-glucose detection at various concentrations (1.0~15.0 mM). (**d**) Stability test compared between the 1st and 14th day. Scan rate for D-glucose detection is from 10 to 100 mV·s^−1^.

The cell potential and power output detection are shown in Figure 4a. The completed redox reactions of immobilized Gox were confirmed. Although Figure 4b,d already shows the stability of the biosensor, the developed biosensor was monitored for 14 days using various glucose concentrations for more than 20 cycles. Figure 4a presents cell potential detection with a freshly established biosensor as well as at 14 days after film electrode was fabricated. The CV results revealed that more than 70% of the current peak remained after 14 days of electrode establishment. Comparing the biosensor EIS performance with a fresh modified electrode in Figure 3c, the redox reactions were all completed by the biosensor with a modified electrode after 14 days (Figure 3d). The performances of both electrodes, fresh and modified 14 days, were investigated with the same D-glucose concentrations. Further, the current values of reaction for these two modified electrodes were also almost the same, as shown in Figure 3c,d. These comparing results indicate the performance of the biosensor, which was maintained after 14 days of electrode modification. The additional redox reaction picks were represented by the after 14-day modified electrode of the biosensor, which still indicated that the sensor effect might be decreased with long-term storage after modification of the electrode. The CV determination of the day-14 biosensor was carried out in a PBS solution with different glucose concentrations. The investigation of this nano-biosensor capacity started from comparing the results of GO/Co/chitosan immobilized electrode (Figure 4a) with the bare Au electrode (Figure 4b). After confirming the modification effects, continuous real-time detection of D-glucose was made with various concentrations, as represented in Figure 4c. Finally, the stability of this biosensor was evaluated by comparing the performance of the film electrode with fresh modification and at 14 days after modification (Figure 4d). Regarding the results shown in Figure 4b, the redox reactions involving the immobilized Gox were completed with 1 to 15 mM of glucose concentration, showing “duck”-shaped CV curves. More importantly, the CV results for different concentrations of glucose could be distinguished, as shown in Figure 4b, indicating the feasibility of glucose sensing at 14 days after the device was established. Such good stability of this biosensor suggests that the developed film-type electrode could be used in electrical devices with long-term operation. Biosensors have been developed for their reliable, rapid, and precise information on food safety [26]. To evaluate the sensing ability, a commercial beverage was selected as a target sample to demonstrate the performance of the biosensor using a film-type electrode for detecting glucose. Functional drinks have been reported to have less sugar content. Thus, they are more suitable than soft drinks and fruit juices for people who need energy supplements and glucose control [27,28,29].

According to the official composition table, sport beverages have various compositions, such as multiple sugars, citrus flavor, citric acid, sodium citrate, sodium chloride, potassium chloride malic acid, calcium lactate, and glucono-delta-lactone, which can influence glucose sensing [30,31,32]. With an increasing scan rate from 0.01 to 0.1 mV/s, the current peak is distinctively presented in Figure 3c,d, indicating that glucose sensing with immobilized Gox could be performed without negligible influences of other compositions. This exclusivity for glucose sensing presented in this demonstration could be considered as the feasibility of using the biosensor developed in this study for simple and fast glucose sensing. Comparing the data of biosensor performance with fresh modification and 14 days after modification of the electrode was investigated, as shown in Figure 4d. The coefficient parameter (R2) values in both electrodes were more than 0.99, which indicated a stable performance maintained after 14 days of electrode modification. Additionally, in previous EFC system work, the GO/Co/chitosan-modified film-type electrode could be used for effective and portable electrical devices [14]. The amperometric response of the GO/Co/chitosan with the Au electrode was obtained via the successive addition of glucose in a continuously stirred 0.1 M PBS (pH 7.0) at a potential of 0.6 V, as shown in Figure 4c. Table 1 shows the comparison of sensor properties among various electrochemical biosensors with the modified electrode with a glucose oxidase (Go)/electron transfer mediator. Our developed biosensor with GO/Co/chitosan had the highest sensitivity among other biosensors, and it was also excellent when comparing linear range and limited detection [33,34,35,36].

## 4. Conclusions

An Au film electrode was created with immobilized Gox on an EDC/NHS bonding GO/Co/chitosan-modified surface to establish a glucose biosensor. The response of the Au−Gox electrode in the absence of the Gox enzyme was significantly suppressed compared to the response in the presence of the enzyme, suggesting that a hydrogen peroxide pathway dominated our Au−Gox electrode. Modification of the GO/Co/chitosan on the surface of the Au film electrode was analyzed by SEM and FT-IR. The hydrogen peroxide pathway of electron transfer dominated in immobilized Gox, which was confirmed by a significantly facilitated CV response compared to the bare electrode. Based on the CV responses of the anodic peak current, the linear detection range of glucose concentration was found to be between 1 and 15 mM. The sensitivity of this glucose biosensor was determined to be 0.14 μA/mM‧cm^2^, with a detection limit of 2.7 mM. The stability of the modified electrode was confirmed. Glucose concentrations could still be distinguished at 14 days after the biosensor was established. Additionally, practical glucose detection for a commercial beverage was demonstrated with this developed film-type biosensor. The results of this study suggest that the Gox-based Au-film working electrode is a promising candidate for a miniaturized glucose biosensor.

## Figures and Tables

**Table 1:** Comparison of sensor properties among various electrochemical biosensors with the modified electrode with glucose oxidase (Go)/electron transfer mediator.

Electrode Materials	Sensitivity (μA mM^−1^ cm^−2^)	Linear Range (mM)	Limit of Detection (mM)	Reference
Go/Co/chitosan	0.14	1.0–15	2.7	This work
Go/chitosan/GCE	0.233	0.6–2.8	1.0	[32]
Graphene-Cds-Go/GCE	1.76	2.0–16	7.0	[33]
Go/PEDOT	8.5	0.5–15	6.5	[34]
CuO nanowires Non-enzyme	1.2	Up to 1	1.0	[36]

GCE: glassy carbon electrode; Cds: cadmium sulfide nanoparticles.

## Data Availability

Not applicable.
